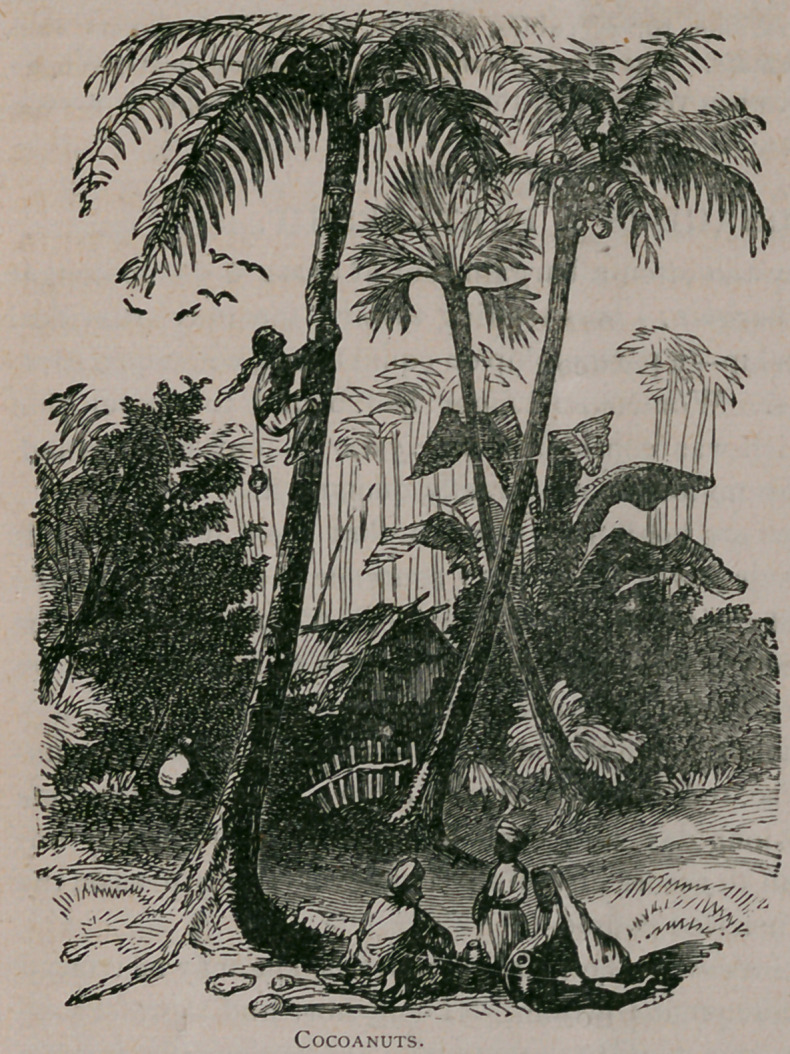# The Cocoanut Tree

**Published:** 1888-01

**Authors:** 


### THE COCOANUT TREE.

Cocoanuts are the fruit of the *Cocos nucifera*, or cocoanut palm, a lofty and elegant palm tree which grows abundantly in most tropical countries; it is from fifty to sixty feet in height, its simple, column-like stem being crowned with a beautiful plume of feathery leaves from twelve to fourteen feet long. The nuts grow in several long clusters depending from the base of the leaves; they are about the size of a man's head, the thin outer rind covering a large mass of fibres, which are used in many countries for the making of mats, cordage, and coarse sail cloth. Within this fibrous coating is the shell of the nut, which is oval and very hard, and often serves for a drinking cup. The kernel is firm, white and pleasant; the interior hollow, and filled with sweet milky juice; when unripe, it is entirely filled with this juice. The cocoanut palm abounds in the East Indies throughout the tropical islands of the Pacific, and also in the West Indies and South America. On the Malabar and Coromandel coasts of India immense groves may be seen. In Ceylon, which is peculiarly well suited for their cultivation, it is estimated that twenty millions of these trees are growing. Here it stands at the head of all trees in its usefulness to man, every particle of stem, leaves and fruit being put to use, and the Cingalese love to repeat to strangers the hundred uses to which they apply it. The following are only a few of the countless uses of this invaluable tree: The *leaves*, for roofing for mats, for baskets, torches or chules, fuel, brooms, fodder for cattle, manure. The *stem of the leaf*, for fences, for pingoes (or yokes) for carrying burdens on the shoulders, for fishing-rods, and innumerable domestic utensils. The *cabbage*, or cluster of unexpanded leaves, for pickles and preserves. The *sap*, for toddy, for distilling arrack, and for making vinegar and sugar. The *unformed nut*, for medicine and sweetmeats. The *young nut* and its milk, for drinking for dessert; the *green husk*, for preserves.

COCOANUTS.

The *nut* for eating, for curry, for milk, for cooking. The *oil*, for rheumatism, for annointing the hair, for soap, for candles, for light : and the *poonak*, or refuse of the nut, after expressing the oil, for cattle and poultry. The *shell of the nut*, for drinking-cups, charcoal, tooth powder, spoons, medicine, hookahs, beads, bottles and knife handles. The *coir*, or fibre which envelopes the shell within the outer husk, for mattresses, cushions, ropes, cables, cordage, canvas, fishing-nets, fuel, brushes, oakum and floor mats. The *trunk*, for rafters, laths, railing, boats, troughs, furniture, firewood ; and when very young, the first shoots or cabbage, as a vegetable for the table.

## Figures and Tables

**Figure f1:**